# High Satisfaction, Modest Differences: A Comparative Cohort Study of Robotic-Assisted and Conventional Total Hip Arthroplasty

**DOI:** 10.3390/jcm15197721

**Published:** 2026-10-07

**Authors:** David González-Martín, Alejandro Herrera-Rodríguez, José María Lomo-Garrote, Juan José González-Álvarez, Francisco Del Canto-Iglesias, Andrés Saldaña-Díaz, José Luis Pais-Brito

**Affiliations:** 1 Department of Orthopaedic Surgery, Hospital Recoletas Salud Campo Grande, 47007 Valladolid, Spain; 2 Facultad de Ciencias de la Salud, Universidad Europea Miguel de Cervantes, 47012 Valladolid, Spain; 3 Department of Orthopaedic Surgery, Hospital Universitario de Canarias, 38320 Tenerife, Spain; 4 Facultad de Ciencias de la Salud, Universidad de La Laguna, 38200 San Cristóbal de La Laguna, Spain; 5 Department of Orthopaedic Surgery, Hospital Clínico Universitario de Valladolid, 47003 Valladolid, Spain

**Keywords:** robotic-assisted total hip arthroplasty, patient-reported outcome measures, patient satisfaction, MAKO

## Abstract

**Background/Objectives:** Robotic-assisted total hip arthroplasty (raTHA) improves the accuracy and reproducibility of acetabular component positioning compared with conventional THA (cTHA), but whether this translates into superior patient-reported outcomes and satisfaction remains debated. **Methods:** A retrospective comparative cohort study was performed, including all patients who underwent primary raTHA with the Mako system at our institution (September 2024–May 2025) and an equal-sized control group recruited consecutively from an alphabetically ordered list of 172 eligible cTHAs, starting from a randomly selected letter (August 2022–January 2025), with a minimum follow-up of one year. Treatment allocation was determined by insurance coverage. The postoperative Western Ontario and McMaster Universities Osteoarthritis Index (WOMAC) was the primary outcome; the Visual Analog Scale (VAS), modified Harris Hip Score (mHHS) and Forgotten Joint Score-12 (FJS-12) were secondary outcomes, and an overall satisfaction rating and willingness to undergo the surgery again were also recorded. **Results:** Ninety-three raTHA and 93 cTHA patients were analyzed. Follow-up was longer in the cTHA group (35.6 vs. 15.5 months; *p* < 0.001). Postoperative WOMAC (10.5 vs. 14.4; mean difference −3.8 points, 95% CI −7.4 to −0.2) and mHHS (86.9 vs. 84.7; mean difference 2.2 points, 95% CI −0.9 to 5.4) differed in rank-based comparisons, favoring raTHA (Mann–Whitney U test, *p* = 0.020 and *p* = 0.030, respectively). Point estimates were below the selected clinical-relevance thresholds, but the confidence intervals did not exclude clinically relevant differences; improvement from baseline did not differ between groups. There were no significant differences in VAS, FJS-12, overall satisfaction (9.32 vs. 9.31) or willingness to undergo surgery again (96.8% vs. 97.8%). **Conclusions:** Given insurance-based allocation, unequal follow-up and unadjusted analyses, robotic-assisted THA was associated with small differences in postoperative WOMAC and mHHS, whose point estimates were below the selected thresholds, although clinically relevant differences cannot be excluded; there were no differences in improvement, pain, joint awareness or satisfaction, which was very high in both groups. These findings do not establish a causal effect of robotic assistance; prospective randomized studies are needed.

## 1. Introduction

Total hip arthroplasty (THA) is among the most successful interventions in orthopedic surgery, yet implant durability and clinical outcome remain closely tied to the precision of component positioning [1]. Conventional manual instrumentation relies on anatomical landmarks and surgeon experience, and a substantial proportion of acetabular components are placed outside recognized safe zones even in experienced hands [1,2]. This has driven increasing adoption of robotic-assisted total hip arthroplasty (raTHA), which has evolved from early autonomous, active systems to contemporary semi-active, haptic-guided platforms such as the Mako system (Stryker, Kalamazoo, MI, USA) [2]. Nationwide projections suggest that robotic assistance will account for the majority of primary THA procedures performed in the United States within the next several years [3].

Multiple systematic reviews, meta-analyses, and an umbrella review of meta-analyses have confirmed that raTHA improves the accuracy and reproducibility of acetabular component positioning, leg-length restoration, and center-of-rotation reconstruction compared with conventional THA (cTHA) [4,5,6]. However, whether this radiographic advantage translates into superior patient-reported outcomes remains debated: several meta-analyses report statistically significant, but not consistently clinically meaningful, differences in functional scores such as the Harris Hip Score (HHS), the Western Ontario and McMaster Universities Osteoarthritis Index (WOMAC), and the Forgotten Joint Score-12 (FJS-12) between raTHA and cTHA [7,8,9]. The distinction between statistical significance and the minimal clinically important difference (MCID) is central to interpreting this literature, since statistically detectable improvements frequently fail to exceed thresholds considered meaningful to patients [10]. Comparative data on patient satisfaction and experience are even more limited, and the available evidence suggests that superior component positioning does not necessarily translate into greater satisfaction [11,12].

Given this uncertainty, the purpose of this study was to compare PROMs and PREMs, including pain, function, and satisfaction, between raTHA and cTHA in a large single-center cohort with a minimum follow-up of one year. We hypothesized that raTHA would be associated with better functional PROMs than cTHA, but not necessarily with higher patient satisfaction, given the multifactorial determinants of these subjective measures.

## 2. Materials and Methods

### 2.1. Study Design and Patients

This study was conducted in accordance with the principles of the Declaration of Helsinki. It was approved by the Clinical Research Ethics Committee (CEIm) of the Valladolid Health Areas (protocol code PI-26-128-O), and informed consent was obtained from all participants. A retrospective comparative cohort study was conducted, reported in accordance with the STROBE guidelines. Robotic-assisted THAs were performed in privately insured patients from the implementation of the Mako system (Stryker, Kalamazoo, MI, USA) at our institution (first procedure 10 September 2024; last 5 May 2025). Conventional THAs were performed in publicly insured patients referred from the public health system waiting list under an agreement with our hospital (first procedure 4 August 2022; last 31 January 2025). The inclusion periods therefore partially overlapped (September 2024 to January 2025). Inclusion criteria were primary THA for end-stage hip osteoarthritis with a minimum follow-up of one year. No patient had undergone previous surgery on the index hip, no bilateral (simultaneous or staged) procedures were performed, and no eligible patient was excluded.

The hospital’s electronic clinical records were reviewed to identify eligible patients, who were then contacted as described below. All postoperative assessments were conducted during a single telephone interview at final follow-up, using a standardized structured interview administered by an independent interviewer who was not involved in the surgical procedures, which ensured identical administration of all instruments in both groups.

Preoperative and postoperative Visual Analog Scale (VAS), modified Harris Hip Score (mHHS), Western Ontario and McMaster Universities Osteoarthritis Index (WOMAC), and Forgotten Joint Score-12 (FJS-12) were compared between groups. During the conventional inclusion period, 172 patients underwent primary cTHA and were eligible as controls. To obtain a control group of equal size to the raTHA cohort (*n* = 93), eligible cTHA patients were listed in alphabetical order by surname, a starting letter was randomly selected, and patients were then consecutively invited in alphabetical order until 93 participants were included (consecutive recruitment from an alphabetical list with a random starting letter). Two patients declined to participate and were replaced by the next patient on the list. No individual matching was performed; the comparability of baseline characteristics between groups was assessed afterwards (Table 1 and Table 2). In addition, all patients were asked to provide an overall rating of the procedure and whether, based on their overall experience, they would undergo the surgery again. Preoperative scores for all instruments were collected prospectively during routine preoperative consultation and retrieved from the clinical records. The mHHS comprises the pain and function domains of the original Harris Hip Score and was scored on its original 0–91 scale, with higher scores indicating better outcomes; no multiplier was applied. The WOMAC was scored from 0 to 96, with lower scores indicating better outcomes. The FJS-12 is scored from 0 to 100, with higher scores indicating less awareness of the hip joint in daily life and therefore a better outcome; preoperatively, it was administered with reference to the native symptomatic hip. All participants completed all items of every instrument, and there were no missing or inapplicable responses.

**Table 1:** Demographic and perioperative characteristics of the cTHA and raTHA groups.

Variable	cTHA (*n* = 93)	raTHA (*n* = 93)	*p* Value
Age, years—mean (range; ±SD)	70.01 (49–86; ±9.70)	68.85 (42–92; ±10.42)	0.562
Sex, *n*/N (%)	Male 56/93 (60.2%); Female 37/93 (39.8%)	Male 53/93 (57.0%); Female 40/93 (43.0%)	0.655
Side, *n*/N (%)	Right 52/93 (55.9%); Left 41/93 (44.1%)	Right 39/93 (41.9%); Left 54/93 (58.1%)	0.057
Hospital stay, days—mean (range; ±SD)	2.29 (2–13; ±1.23)	2.01 (1–3; ±0.28)	0.137
Implant complications, *n*/N (%)	4/93 (4.3%)	3/93 (3.2%)	1.000
Revision surgery, *n*/N (%)	4/93 (4.3%)	3/93 (3.2%)	1.000
**Follow-up, months—mean (range; ±SD)**	**35.6 (16.7–46.6; ±8.9)**	**15.5 (12.1–21.4; ±2.7)**	**<0.001**

cTHA, conventional total hip arthroplasty; raTHA, robotic-assisted total hip arthroplasty; SD, standard deviation. Values are mean (range; ±SD) or *n*/N (%). Continuous variables were compared with the Mann–Whitney U test and proportions with the chi-square test or Fisher’s exact test, as appropriate. Bold values indicate statistically significant differences (*p* < 0.05).

**Table 2:** Baseline standardized mean differences and between-group effect sizes.

Variable	Baseline SMD	Postoperative/Overall Difference (95% CI)	Change-Score Difference (95% CI)
Age	0.12	—	—
Sex (male)	0.07	—	—
Side (right)	0.28	—	—
VAS (0–10)	0.11	−0.18 (−0.62 to 0.26)	−0.03 (−0.65 to 0.59)
mHHS (0–91)	0.19	2.24 (−0.91 to 5.39)	0.37 (−3.91 to 4.65)
WOMAC (0–96)	0.04	−3.83 (−7.45 to −0.21)	−3.48 (−7.98 to 1.02)
FJS-12 (0–100)	0.03	−0.11 (−3.31 to 3.09)	0.30 (−5.32 to 5.92)
Overall satisfaction rating (0–10)	—	0.01 (−0.39 to 0.41)	—
Would undergo surgery again	—	−1.1% (−5.7 to 3.6)	—
Implant complications	—	−1.1% (−6.5 to 4.4)	—
Revision surgery	—	−1.1% (−6.5 to 4.4)	—

Differences are raTHA minus cTHA: mean differences for continuous variables and risk differences for proportions. For VAS and WOMAC, lower scores indicate better outcomes, so negative differences favor raTHA; for mHHS, FJS-12 and satisfaction, positive differences favor raTHA. *p* values in Table 3 refer to the Mann–Whitney U test (rank-based); 95% CIs refer to differences in means (Student t distribution) or risk differences (Wald method). SMD, standardized mean difference (absolute value); CI, confidence interval; other abbreviations as in Table 1 and Table 3.

**Table 3:** Patient-reported outcome measures (PROMs) and patient-reported experience measures (PREMs).

Variable	cTHA (*n* = 93)	raTHA (*n* = 93)	*p* Value
**VAS (0–10)**	—	—	—
Preoperative	8.84 (3–10; ±1.35)	8.69 (3–10; ±1.35)	0.311
Postoperative	0.70 (0–7; ±1.57)	0.52 (0–7; ±1.49)	0.340
Δ score	−8.14 (−10–1; ±2.18)	−8.17 (−10–2; ±2.07)	0.642
**mHHS (0–91)**	—	—	—
Preoperative	29.68 (15–69; ±10.15)	31.56 (16–74; ±9.52)	0.141
**Postoperative**	**84.68 (34–91; ±11.20)**	**86.92 (18–91; ±10.59)**	**0.030**
Δ score	55.00 (2–76; ±14.93)	55.37 (−16–75; ±14.62)	0.731
**WOMAC (0–96)**	—	—	—
Preoperative	79.22 (48–92; ±8.65)	78.87 (43–93; ±8.84)	0.773
**Postoperative**	**14.37 (0–62; ±13.54)**	**10.54 (0–81; ±11.35)**	**0.020**
Δ score	−64.85 (−88–−18; ±15.75)	−68.33 (−90–7; ±15.35)	0.073
**FJS-12 (0–100)**	—	—	—
Preoperative	15.74 (0–91; ±15.84)	15.34 (0–91; ±15.4)	0.845
Postoperative	78.67 (24–94; ±10.59)	78.56 (12–93; ±11.52)	0.832
Δ score	62.92 (−16–88; ±18.77)	63.22 (−24–89; ±20.04)	0.680
Overall satisfaction rating (0–10)—mean (range; ±SD)	9.31 (3–10; ±1.33)	9.32 (0–10; ±1.46)	0.883
Would undergo surgery again, *n*/N (%)	91/93 (97.8%)	90/93 (96.8%)	1.000

VAS, Visual Analog Scale; mHHS, modified Harris Hip Score; WOMAC, Western Ontario and McMaster Universities Osteoarthritis Index; FJS-12, Forgotten Joint Score-12; cTHA, conventional total hip arthroplasty; raTHA, robotic-assisted total hip arthroplasty; SD, standard deviation. Bold values indicate statistically significant differences (*p* < 0.05). Values are mean (range; ±SD) or *n*/N (%). Continuous variables were compared with the Mann–Whitney U test and proportions with Fisher’s exact test. All results are based on 93 patients per group.

All surgeries were performed by orthopedic surgeons with high-volume experience, each performing more than 50 THAs per year. Assignment to raTHA or cTHA was not randomized: because it was determined by the patient’s insurance coverage rather than by treatment allocation, a selection bias affecting both treatment assignment and survey compliance cannot be excluded. The raTHA group had private insurance, and the cTHA group had public health insurance. All surgeries were performed by the same surgical team, regardless of the patients’ insurance coverage. Insurance status may be associated with socioeconomic factors, comorbidities, access to care and patient expectations, and is therefore a potential source of residual confounding. The allocation rule was strictly administrative: all privately insured patients underwent raTHA without exception, no publicly insured patient had access to robotic surgery, and no clinical or radiographic criteria (such as developmental dysplasia, prior hip surgery, retained hardware, body mass index or contraindications to CT) were used to select patients for either technique.

All procedures were performed at the same hospital (Hospital Recoletas Salud Campo Grande, Valladolid, Spain), and all patients followed an identical perioperative pathway, including preoperative assessment, surgical preparation, anesthesia, antibiotic and thromboembolic prophylaxis, postoperative rehabilitation and follow-up. The only difference was the preoperative CT scan required for robotic planning in the raTHA group.

### 2.2. Surgical Technique

All surgeries were performed under spinal anesthesia using a modification of the posterolateral approach known as the superior transverse atraumatic reconstruction (STAR) technique, which preserves piriformis integrity and minimizes soft-tissue disruption while maintaining the extensile exposure and visualization of the conventional posterolateral approach [13].

In both groups, an uncemented Accolade II femoral stem, a Trident X3 acetabular cup, and a BIOLOX delta ceramic V40 femoral head (Stryker, Kalamazoo, MI, USA) were used. Liner selection followed a fixed institutional protocol rather than individual patient characteristics. In conventional THA, a 10° lipped liner was routinely used to reduce the risk of dislocation. Following implementation of the Mako system, a neutral liner was used in all raTHA cases, based on the greater confidence in component positioning afforded by robotic planning and execution, to avoid potential impingement associated with the elevated rim. Local intraoperative anesthesia was administered at the end of surgery in all cases.

The robotic system (MAKO^®^; Stryker, Kalamazoo, MI, USA) was calibrated and configured following a standardized protocol before surgery. Preoperative computed tomography (CT) scans of the hip, knee, and ankle were obtained to build a patient-specific three-dimensional model using the THA application platform, which was used to plan implant size and position preoperatively. Intraoperatively, tracking arrays were fixed to the cup and femur, and bone registration was performed before proceeding with robotic-assisted bone resection.

### 2.3. Statistical Analysis

As this was a retrospective study, no primary outcome was formally prespecified. For the purposes of this analysis, the postoperative WOMAC total score at final follow-up was considered the primary outcome, as it is the most comprehensive of the instruments used, capturing pain, stiffness and function. Postoperative and change scores for the VAS, mHHS and FJS-12 were considered secondary outcomes, and satisfaction, willingness to undergo surgery again, complications and revisions were considered exploratory.

The normality of continuous variables was assessed using the Shapiro–Wilk test. As the distributions of VAS, mHHS and WOMAC scores deviated significantly from normality (*p* < 0.001), with marked ceiling and floor effects, all between-group comparisons of continuous and ordinal variables were performed using the Mann–Whitney U test. *p* values for continuous and ordinal outcomes refer to this rank-based comparison of the distributions in the two groups. Categorical variables were compared using the chi-square test or, when any expected cell count was below five, Fisher’s exact test. Effect sizes are reported as mean differences (raTHA minus cTHA) with 95% confidence intervals (CIs) for continuous outcomes and as risk differences with 95% CIs for proportions. Confidence intervals refer to the difference in means and were calculated using the Student t distribution (for risk differences, the Wald method); they estimate the magnitude and precision of the mean difference and were not used to establish a non-zero mean advantage. Because the data were skewed with ceiling effects, the rank-based test and the interval for the mean difference may lead to different conclusions. Baseline comparability was additionally assessed using standardized mean differences (SMDs), with values below 0.1 indicating negligible imbalance. Baseline *p* values were not used to infer comparability, and no covariate-adjusted analyses were performed.

The clinical relevance of the results was interpreted using two approaches. For within-patient improvement, published anchor-based minimal clinically important difference (MCID) values for patients undergoing THA were used: 18.6 mm for VAS pain [14], 16–18 points for the Harris Hip Score [15], applied to the mHHS as no validated anchor-based threshold specific to this version was available for THA, 8.3 points on a 0–100 scale for WOMAC, the lowest estimate reported for THA [16] (applied unchanged to the 0–96 score used here; the proportionally equivalent value, 8.0 points, would not alter any interpretation), and 17.5 points for FJS-12 [17]. Because within-patient MCIDs are not intended to interpret differences in mean scores between groups, between-group differences were additionally interpreted using a distribution-based threshold of half the pooled baseline standard deviation [18], corresponding to 0.7 points for VAS, 4.9 for mHHS, 4.4 for WOMAC and 7.8 for FJS-12. This half-standard-deviation criterion is an interpretive benchmark rather than a validated patient-anchored threshold; we therefore compared the limits of the 95% CIs of each between-group difference with this benchmark to judge whether clinically relevant differences could be excluded.

All 186 patients completed all instruments and there were no missing data; every result is therefore based on 93 patients per group. No adjustment for multiple comparisons was applied to secondary and exploratory outcomes, which should be interpreted as hypothesis-generating. A non-significant result was not interpreted as evidence of equivalence. No a priori sample size calculation was performed; all patients who underwent raTHA during the study period were included, and a control group of equal size was selected as described above. Probability values of less than 0.05 were considered significant. Data analyses were performed using the SPSS statistical package (IBM Corp. Released 2017. IBM SPSS Statistics for Windows, Version 25.0, Armonk, NY, USA). All tabulated values were verified against the source database and, for the mHHS, the original questionnaires before final analysis; corrections were limited to transcription and data-entry errors, and the analyzed cohort (93 patients per group) was unchanged.

## 3. Results

**Figure 1:**
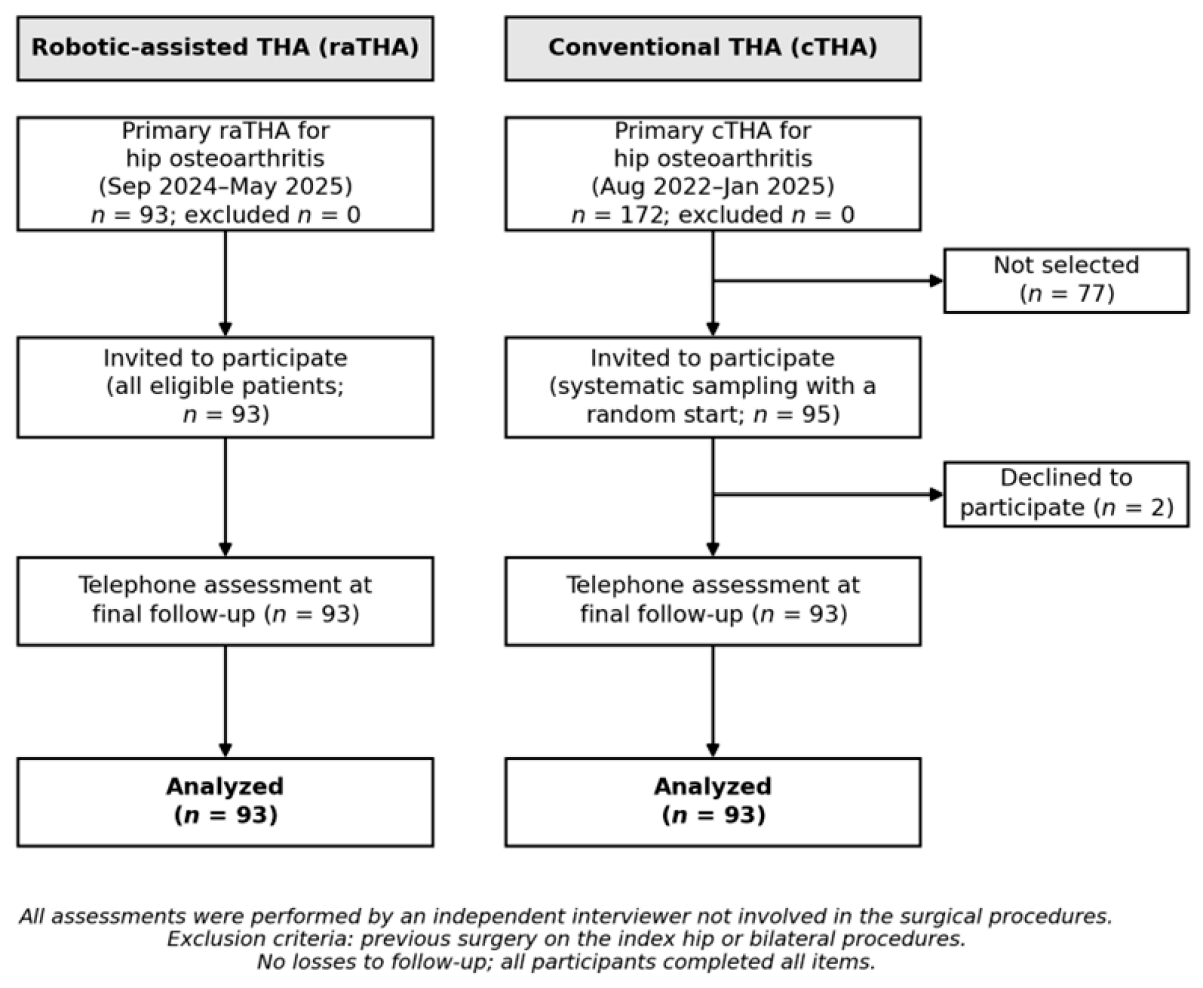
STROBE flow diagram of participant selection.

During the study period, 93 raTHAs and 172 cTHAs were performed. All 93 raTHA patients were included, with no losses to follow-up. Of the 172 eligible cTHA patients, 95 were invited following the selection procedure described above; two declined to participate, leaving 93 cTHA patients for analysis (Figure 1). There were no significant differences between the groups in terms of demographics, hospital stay, implant complications or revision surgery rate, although standardized differences indicated modest imbalances in age and side (Table 2). Follow-up was significantly longer in the cTHA group (35.6 ± 8.9 vs. 15.5 ± 2.7 months; *p* < 0.001) (Table 1).

Postoperative WOMAC scores, the primary outcome, differed between groups in the rank-based comparison, favoring raTHA (10.54 ± 11.35 vs. 14.37 ± 13.54; Mann–Whitney U test, *p* = 0.020; mean difference −3.8 points, 95% CI −7.4 to −0.2). Postoperative mHHS scores also differed in the rank-based comparison (86.92 ± 10.59 vs. 84.68 ± 11.20; Mann–Whitney U test, *p* = 0.030), whereas the 95% CI of the mean difference (2.2 points; −0.9 to 5.4) included zero. These results address different quantities and are not contradictory: the Mann–Whitney test compares rank distributions, whereas the confidence interval refers to the difference in means, which is more sensitive to extreme values and to the ceiling effect. However, the medians of both scores were similar (WOMAC 7 vs. 9; mHHS 91 in both groups), the point estimates of these differences were below the MCID and distribution-based thresholds described in the Methods (although the confidence intervals included values exceeding the distribution-based thresholds), and the change from baseline did not differ significantly between groups for either WOMAC (*p* = 0.073) or mHHS (*p* = 0.731). No significant differences were observed in preoperative, postoperative or change scores for VAS or FJS-12. The mean overall satisfaction rating was 9.31 (range 3–10; ±1.33) in the cTHA group and 9.32 (range 0–10; ±1.46) in the raTHA group (*p* = 0.883), and 91/93 (97.8%) and 90/93 (96.8%) patients, respectively, stated that they would undergo the surgery again (*p* = 1.000) (Table 3). Baseline standardized mean differences and between-group effect sizes are shown in Table 2. Baseline standardized mean differences exceeded 0.1 for age (0.12), side (0.28), VAS (0.11) and mHHS (0.19); baseline mHHS was slightly higher in the raTHA group (31.56 vs. 29.68).

There were 4 implant complications in the cTHA group: femoral stem loosening (stem revision), dislocation (dual mobility hip revision), intraoperative periprosthetic acetabular fracture (acetabular revision) and infection (DAIR). There were 3 implant complications in the raTHA group, all infections: two were treated with DAIR and one with two-stage revision surgery. Two of these were acute postoperative infections and one was a late infection; none was related to the tracking array or pin sites. There were no significant differences between groups (*p* = 1.000). There were no medical complications or pin-site fractures.

## 4. Discussion

In this retrospective comparative study of 186 patients, raTHA using the Mako system was associated with postoperative WOMAC and mHHS scores that favored raTHA over cTHA at final follow-up in rank-based comparisons. However, the point estimates of these differences (3.8 and 2.2 points, respectively) were below both anchor-based MCID values and distribution-based thresholds for between-group differences, although the 95% confidence intervals of both the WOMAC (−7.4 to −0.2) and the mHHS (−0.9 to 5.4; the interval also included zero) differences included values exceeding the distribution-based thresholds (4.4 and 4.9 points, respectively), so clinically relevant between-group differences cannot be definitively excluded for either outcome. The half-standard-deviation criterion is an interpretive benchmark rather than a validated patient-anchored threshold. Improvement from baseline did not differ between groups. No significant differences were observed in pain, joint awareness, overall satisfaction or willingness to undergo the surgery again. Both groups reported very high satisfaction, with more than 96% of patients in each group stating that they would undergo the surgery again, and complication and revision rates did not differ significantly between groups, although the rate of periprosthetic joint infection in the raTHA group was higher than expected, as discussed below. These findings derive from a non-randomized comparison in which treatment allocation was determined by insurance coverage and follow-up differed substantially between groups (35.6 vs. 15.5 months); both factors, together with the absence of adjusted analyses, preclude attributing the observed differences to robotic assistance.

These findings are consistent with the highest-quality comparative evidence currently available. The largest meta-analysis published to date, pooling 38 studies and more than 10,000 patients, found no significant difference in HHS, WOMAC, FJS-12, or Merle d’Aubigné scores between raTHA and cTHA despite raTHA’s superior radiological accuracy [7]. Similarly, a meta-analysis restricted to Mako-specific cohorts found that although FJS-12 significantly favored raTHA, the improvement did not exceed the MCID [19], and a systematic review specifically assessing MCID achievement across ten robotic-assisted THA studies found that although significant improvements were reported in 60% of studies, none reached a clinically important relative improvement [10]. Our WOMAC and mHHS findings follow a similar pattern: small differences favoring raTHA, which reached statistical significance only in rank-based comparisons, of uncertain clinical relevance. Comparative satisfaction data remain particularly scarce in the literature; a pair-matched study similarly found no difference in satisfaction despite superior HHS and FJS-12 scores with raTHA [11], and a patient stakeholder survey found broadly favorable perceptions of raTHA even among patients unfamiliar with the technology, without directly measuring comparative postoperative satisfaction against cTHA [12]. Regarding safety, the complication and revision rates observed in both groups are in keeping with pooled data from an umbrella review reporting reduced overall complications with raTHA without a corresponding reduction in revision risk at the highest level of evidence [4], and with a fragility analysis of randomized trials showing that revision-risk comparisons in this literature remain statistically fragile [20]. However, our study was not powered to detect differences in these low-frequency outcomes.

Beyond these quantitative outcomes, robotic assistance may also confer a potential benefit for the surgeon: it provides the surgeon with a degree of intraoperative reassurance that the final radiographic result will closely reproduce the preoperative plan, since the haptic boundary constrains bone preparation and component insertion in real time [2,21]. In conventional surgery, by contrast, the radiographic outcome, although frequently very similar to the intended plan, is achieved through anatomical landmarks and surgeon judgment alone, and a perfectly reproduced plan is not always obtained even in experienced hands [1,2]. This difference may partly explain why surgeons increasingly favor robotic assistance despite its inconsistent translation into superior patient-reported outcomes. From the patient’s perspective, however, satisfaction was very high in both groups: the median rating was 10 in both, and two-thirds of patients in each group gave the maximum score. Similar ceiling and floor effects were observed for other PROMs, with 77% of cTHA and 83% of raTHA patients reporting no pain, and 52% and 66%, respectively, achieving the maximum mHHS score. Such ceiling effects, previously highlighted as a major methodological obstacle to establishing the added clinical value of robotic systems [2,22], limit the ability of these instruments to discriminate between groups. The absence of differences in satisfaction should therefore be interpreted as a limitation of the measures used rather than as evidence that both techniques are equivalent from the patient’s perspective.

From a health-economic perspective, robotic assistance carries higher upfront capital and per-case costs, which may be partly offset by shorter hospital stays and fewer readmissions in high-volume centers [23,24]; in the present cohort, hospital stay did not differ significantly between groups, which may reflect the mature, high-volume practice of both surgical pathways at our institution.

The rate of periprosthetic joint infection in the raTHA group (3/93, 3.2%; 95% CI 1.1–9.1%) was higher than the approximately 1–2% reported after primary THA [25], whereas one infection occurred in the cTHA group (1.1%; *p* = 0.621), despite its considerably longer follow-up. Two of the raTHA infections were acute postoperative infections and one was a late infection, and none were related to the tracking array or pin sites. Given the small number of events, this finding may reflect chance, and a recent meta-analysis did not find an increased risk of periprosthetic joint infection with robotic-assisted THA [26]. Nevertheless, the potentially longer operative time during the early phase of the robotic program and the additional incisions required for tracking arrays cannot be excluded as contributing factors, and operative time, microbiological data and patient-level risk factors were not available for analysis. This finding should be monitored in larger series with longer follow-up.

Taken together, although robotic assistance has consistently been shown to improve the accuracy and reproducibility of component positioning [4,5,6], which was not assessed radiographically in the present study, we did not observe a clear clinically meaningful benefit in patient-reported outcomes or satisfaction at short-term follow-up. This does not exclude potential benefits in other domains, such as long-term implant survivorship, dislocation risk or the management of complex anatomy.

This study has several limitations. Its retrospective design carries an inherent risk of bias. Treatment allocation was determined by insurance coverage: all raTHA patients had private insurance and all cTHA patients had public insurance. Insurance status may be associated with socioeconomic factors, comorbidities, access to care and patient expectations, all of which may influence PROMs and satisfaction. Owing to the retrospective design, data on body mass index, ASA grade, comorbidities and socioeconomic status were not available, and residual confounding cannot be excluded. Although both groups were treated by the same surgical team using identical perioperative, anesthetic and rehabilitation protocols, insurance status is a potential confounder and our findings should be interpreted accordingly. Baseline characteristics were broadly similar, but standardized mean differences exceeded 0.1 for age (0.12), side (0.28), VAS (0.11) and mHHS (0.19). Baseline mHHS was slightly higher in the raTHA group, which could favor raTHA postoperatively, and the postoperative mHHS difference is of similar magnitude to this baseline difference. Non-significant baseline *p* values do not establish comparability, and no covariate-adjusted analyses were performed because key confounders such as comorbidities and socioeconomic status were not available; the postoperative differences should therefore be interpreted as unadjusted. Regarding the likely direction, privately insured patients are likely to have a higher socioeconomic status, which has been associated with greater functional improvement and higher satisfaction after THA [27], and may also have greater health literacy and shorter waiting times; these factors would be expected to bias PROMs in favor of the raTHA group, so the small differences observed in WOMAC and mHHS could be partly or entirely explained by insurance-related confounding rather than by robotic assistance. Conversely, higher expectations among privately insured patients could bias satisfaction against the raTHA group. The magnitude of this bias cannot be quantified with the available data, and the broadly similar preoperative PROMs offer only limited reassurance regarding baseline disease severity. Although PROMs provide critical insight into therapeutic efficacy and subjective recovery, their validity relies on diligent self-reporting, which can be influenced by diverse patient-specific factors. In addition, no a priori sample-size or power calculation was performed, and the cohort was not specifically powered to detect differences in low-frequency events such as implant complications and revision surgery; consequently, the non-significant differences observed for these outcomes should be interpreted with caution, as a true effect of modest magnitude cannot be excluded. Follow-up was significantly longer in the cTHA group (35.6 vs. 15.5 months), because the conventional inclusion period started before the implementation of the robotic system and the inclusion periods only partially overlapped; although all patients exceeded one year of follow-up, by which time PROMs after THA have generally plateaued, the cTHA group had a longer exposure period for complications and revisions. Because PROMs were collected in a single interview at final follow-up, the interval since surgery differed substantially between groups, and the direction and magnitude of any resulting bias in the PROM comparisons cannot be determined from our data. Liner design also differed between groups according to institutional protocol (10° lipped in cTHA, neutral in raTHA); because the elevated rim may influence stability, range of motion and impingement, liner design is a potential confounder, and differences in PROMs or complications cannot be attributed solely to robotic assistance. The control group was recruited consecutively from an alphabetically ordered list with a random starting letter; although this is unrelated to clinical characteristics, it is not a probability sample, and selection bias cannot be fully excluded. The marked ceiling effects observed for satisfaction, pain and mHHS limit the ability of these measures to detect differences between groups. Radiographic parameters of component positioning were not collected, so we cannot confirm that raTHA achieved more accurate positioning in our cohort or relate positioning to PROMs. Finally, operative time, microbiological data and patient-level risk factors for infection were not available.

Despite these limitations, our study offers several strengths compared with previous investigations characterized by smaller cohorts and heterogeneous implant designs: a representative sample size and the use of the same surgical approach and the same implant system in both groups. All procedures, robotic-assisted and conventional alike, were performed through the STAR (superior transverse atraumatic reconstruction) modification of the posterolateral approach, which preserves the piriformis, and all patients received the same implant components (uncemented Accolade II femoral stem, Trident X3 acetabular cup, and BIOLOX delta ceramic V40 femoral head; Stryker), differing only in liner design. The use of the same surgical approach and implant system in both groups reduces the risk of confounding related to differences in surgical exposure or prosthetic selection, although differences in liner design and insurance-based allocation remain potential sources of confounding. We used validated PROMs and PREMs to assess clinical and satisfaction outcomes, analyzed complications and revisions, and reported outcomes for patients with a minimum follow-up of one year.

## 5. Conclusions

In this retrospective, non-randomized comparative cohort, in which treatment allocation was determined by insurance coverage and follow-up was substantially longer in the conventional group (35.6 vs. 15.5 months), raTHA was associated with small differences in postoperative WOMAC and mHHS scores compared with cTHA. The point estimates of these differences were below the selected thresholds of clinical relevance, although the confidence intervals do not exclude clinically relevant differences. No differences were observed in improvement from baseline, pain, joint awareness or patient satisfaction, which was very high in both groups. Because of residual confounding (including insurance status and liner design), modest baseline imbalances and the absence of adjusted analyses, these findings should be interpreted cautiously and do not establish a causal effect of robotic assistance. Prospective randomized studies are needed to clarify the clinical value of robotic assistance in THA.

## Data Availability

The data presented in this study are available on request from the corresponding author due to institutional and patient privacy restrictions.

## Abbreviations

The following abbreviations are used in this manuscript:

raTHA	Robotic-assisted total hip arthroplasty
cTHA	Conventional total hip arthroplasty
PROM	Patient-reported outcome measure
PREM	Patient-reported experience measure
VAS	Visual Analog Scale
mHHS	Modified Harris Hip Score
WOMAC	Western Ontario and McMaster Universities Osteoarthritis Index
FJS-12	Forgotten Joint Score-12
MCID	Minimal clinically important difference
CT	Computed tomography
SD	Standard deviation
DAIR	Debridement, antibiotics, and implant retention
SMD	Standardized mean difference
CI	Confidence interval
